# The impact on selection of non-alcoholic vs alcoholic drink availability: an online experiment

**DOI:** 10.1186/s12889-020-08633-5

**Published:** 2020-05-06

**Authors:** Anna K. M. Blackwell, Katie De-loyde, Gareth J. Hollands, Richard W. Morris, Laura A. Brocklebank, Olivia M. Maynard, Paul C. Fletcher, Theresa M. Marteau, Marcus R. Munafò

**Affiliations:** 1 School of Psychological Science, University of Bristol, 12a Priory Road, Bristol, BS8 1TU UK; 2 Behaviour and Health Research Unit, University of Cambridge, Cambridge, CB2 0SR UK; 3 Bristol Medical School: Population Health Sciences, Canynge Hall, 39 Whatley Road, Bristol, BS8 2PS UK; 4 Department of Psychiatry, University of Cambridge, Forvie Site, Robinson Way, Cambridge, CB2 0SZ UK; 5 The Wellcome-MRC Institute of Metabolic Science-Metabolic Research Laboratories (IMS-MRL), University of Cambridge, Cambridge, CB2 0QQ UK; 6 MRC Integrative Epidemiology Unit (IEU), University of Bristol, Oakfield House, Oakfield Grove, Bristol, BS8 2BN UK; 7 National Institute for Health Research Bristol Biomedical Research Centre (NIHR Bristol BRC), Oakfield House, Oakfield Grove, Bristol, BS8 2BN UK

**Keywords:** Alcohol, Non-alcoholic, Alcohol-free, Public health, Policy, Choice architecture, Availability

## Abstract

**Background:**

Increasing the availability of healthier food increases its selection and consumption. However, there is an absence of evidence related to alcohol. This study aimed to estimate the impact of increasing the absolute and relative availability of non-alcoholic compared to alcoholic drinks on selection. We also assessed whether effects were modified by cognitive resource.

**Methods:**

UK adult weekly alcohol consumers (*n* = 808) were recruited to an online experiment with a hypothetical drink selection task. Participants were randomly assigned to one of eight conditions, in a 4 (availability) × 2 (cognitive resource) factorial design. The four availability conditions were: *i. Reference* 1 (two non-alcoholic, two alcoholic drinks); *ii. Reference* 2 (four non-alcoholic, four alcoholic drinks); *iii*. *Increased non-alcoholic drinks* (six non-alcoholic, two alcoholic drinks); *iv. Increased alcoholic drinks* (two non-alcoholic, six alcoholic drinks). The two cognitive resource conditions were: *a. Low* (high time pressure); *b. High* (low time pressure). Logistic regression was used to assess selection of a non-alcoholic drink.

**Results:**

49% of participants selected a non-alcoholic drink in the *Increased non-alcoholic drinks* condition, compared to 36% in *Reference* 1, 39% in *Reference* 2, and 26% in the *Increased alcoholic drinks* condition. Non-alcoholic drink selection was similar between *Reference 1* and *2* when the total number of drinks increased (absolute availability) but the proportion of non-alcoholic compared to alcoholic drinks (relative availability) was unchanged (OR = 1.15, 95% CI 0.77, 1.73). In contrast, the odds of selecting a non-alcoholic drink were 71% higher when both absolute and relative availability of non-alcoholic compared to alcoholic drinks was increased from *Reference* 1 to the *Increased non-alcoholic drinks* condition (OR: 1.71, 95% CI 1.15, 2.54), and 48% higher when increased from *Reference* 2 to the *Increased non-alcoholic drinks* condition (OR: 1.48, 95% CI 0.99, 2.19). There was no evidence of an effect of cognitive resource.

**Conclusions:**

Greater availability of non-alcoholic drinks, compared to alcoholic drinks, increased their online selection, an effect that may be larger when changing their relative availability, i.e., increasing the proportion of non-alcoholic drinks. Naturalistic studies are needed to determine the impact of availability interventions on reducing alcohol purchasing and consumption.

## Background

Alcohol consumption is associated with over 200 health conditions and is among the top five risk factors for disease globally [1, 2], including in the UK [3]. Excessive alcohol use also creates a substantial burden on public services, including over one million hospital admissions and £3.5 billion in costs to the National Health Service per year [4]. A review of the effectiveness and cost-effectiveness of alcohol control policies compared those that target the three key factors related to harm: affordability, advertising and availability of alcohol [5]. Policies addressing affordability (e.g., increasing taxation) were identified as most successful. Regulating alcohol marketing, particularly reducing exposure to children, and reducing the hours during which alcohol is available, were found to also be effective harm reduction strategies. In comparison, there was very low impact of efforts to remove the overall amount of alcohol sold through improving low-alcohol product options: an agreement made by the alcohol industry as part of a broader voluntary deal with the United Kingdom (UK) government in 2011. The reviewers suggested that the focus on promoting new low-strength options may have instead increased the number of alcohol units on the market [5]. However, the review maintains the importance of implementing a broad approach to harm reduction that combine multiple strategies to maximise their impact.

While efforts to reduce alcohol availability by increasing the choice of low- or non-alcoholic drink options may have had limited effect when attempted at the macro level, increasing these options at the micro level, within the drinking environments that individuals are exposed to, may have more success. Interventions that alter the proximal micro-environments in which behaviours occur – often called ‘choice architecture’ or ‘nudge’ interventions – hold promise for reducing consumption [6]. Interventions that increase the number or range of healthier products in places where they are purchased or consumed, including supermarkets, bars, restaurants, workplaces, can facilitate healthier consumption [7–10]. For example, a stepped wedge randomised controlled pilot trial across six worksite cafeterias in England, found a 6.9% reduction of energy (calories) purchased when a proportion of higher-energy options were replaced with lower-energy options, without impacting revenue [9].

Based on the Typology of Interventions in Proximal Physical Micro-Environments (TIPPME) [6], these interventions are classified as ‘Availability’ interventions that target the product of consumption itself (Availability x Product). They can be further defined according to whether they alter absolute availability (the total number of options)*,* relative availability (the proportion of a subset of options) or a combination of both [11]. A recent Cochrane review of studies examining the impact on selection and consumption of altering the availability or proximity of food, alcohol and tobacco products [10] identified six availability interventions all focused on food. The review findings suggest a large effect on selection and a moderate effect on consumption of reducing the availability of a target category (e.g., less healthy foods), although with considerable uncertainty about the reliability of these effects in part due to studies being limited in quality and quantity. Importantly, no studies were identified that examined availability in relation to selection or consumption of alcohol products [10].

The mechanisms by which availability interventions have their effects are little studied. Possible mechanisms include increased awareness of healthier alternatives, through greater exposure to these options overall, or increasing their comparative visibility to alcohol options, widening the appeal of choices for consumers and encouraging their selection [12]. In addition, greater availability of healthier product alternatives could shift social norms regarding the desirability of their selection and consumption [13], which may be of particular relevance in relation to the strong social pressure to consume alcohol [14, 15].

In real-world settings people are likely to make purchases and consume under conditions of limited cognitive resource. The ecological validity of any intervention is therefore enhanced by assessing its impact under conditions that simulate real-world conditions. The reflective-impulsive model of behaviour [16] proposes that behaviour is controlled by two interacting systems: a reflective system based on knowledge and values and an impulsive system based on associative links and motivation, in which the former can be impaired and the latter more prominent under conditions of limited cognitive resource. Some online product selection studies have included tasks to reduce cognitive resource, for example, digit recall [17], or imposing a time pressure on selection [18, 19]. It is important to assess whether the impact on alcohol-related drink selection of altered product availability is moderated under conditions of reduced cognitive resource, as this could impact on the effectiveness of such interventions in real-world settings.

The current study examined the impact of altering both absolute and relative availability, as well as the range, of non-alcoholic and alcoholic drinks on the selection of a non-alcoholic drink and assessed whether any effects were modified by cognitive resource.

### Hypothesis

Increasing the absolute (total number) and relative (proportion) availability of non-alcoholic drinks increases the likelihood of selecting a non-alcoholic drink compared to an alcoholic drink.

## Methods

### Study design

UK adults who consume alcohol weekly were recruited to an online 4 × 2 factorial experiment, with between-subjects factors of availability (*Reference* 1: two non-alcoholic drinks and two alcoholic beers; *Reference* 2: four non-alcoholic drinks and four alcoholic beers; *Increased non-alcoholic drinks*: six non-alcoholic drinks and two alcoholic beers; *Increased alcoholic drinks*: two non-alcoholic drinks and six alcoholic beers) and cognitive resource (*Low*: high time pressure; *High*: low time pressure). Participants were randomly assigned to one of eight conditions via the Qualtrics online survey platform [20] on which the study was hosted. The study protocol and analysis plan were preregistered [21].

#### Availability of non-alcoholic and alcoholic drinks

**Fig. 1:**
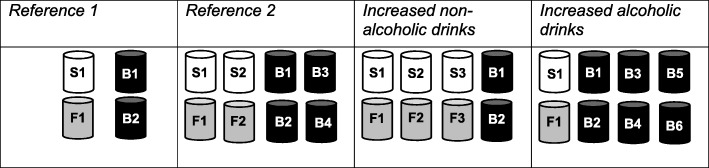
Illustration of the availability of non-alcoholic drinks (S1, S2, S3 = different types of soft drink; F1, F2, F3 = different brands of alcohol-free beer) compared to alcoholic drinks (B1, B2, B3, B4, B5, B6 = different brands of beer) in the four availability conditions

Within their randomised condition, participants were asked to choose one drink that they would like to drink today. All drink selections included two non-alcoholic drinks (one soft drink and one alcohol-free beer) and two brands of alcoholic beers (*Reference* 1) displayed in two rows (Fig. 1). In the second reference condition (*Reference* 2) there were two additional non-alcoholic drinks (one other type of soft drink and one other brand of alcohol-free beer) and two additional brands of alcoholic beers. In the *Increased non-alcoholic drink*s condition, there were four additional non-alcoholic drinks (two other types of soft drink and two other brands of alcohol-free beer). In the *Increased alcoholic drinks* condition, there were four additional brands of alcoholic beers. Alcohol-free beers were clearly labelled as such on the container to avoid any potential confusion with alcoholic beer.

Compared to *Reference* 1: the absolute availability of non-alcoholic drinks was increased in *Reference* 2 (two to four); both the absolute (two to six) and relative (50 to 75%) availability of non-alcoholic drinks was increased in the *Increased non-alcoholic drinks* condition; and the relative availability of non-alcoholic drinks was decreased in the *Increased alcoholic drinks* condition (50 to 25%). In the three comparison conditions, the absolute number of all drinks available was increased (four to eight).

Compared to *Reference* 2: the absolute (four to six) and relative (50 to 75%) availability of non-alcoholic drinks was increased in the *Increased non-alcoholic drinks* condition; and the absolute (four to two) and relative (50 to 25%) availability of non-alcoholic drinks was decreased in the *Increased alcoholic drinks* condition. In the two comparison conditions, the absolute number of all drinks available was kept constant (eight).

Similar hypothetical drink selection tasks have been used previously for sugar sweetened beverages [22–24] and snacks [17].

#### Cognitive resource

This study used a time pressure manipulation to reduce cognitive resource, to ensure the manipulation was implemented reliably in the online setting. Participants were given either eight seconds (*High time pressure*: low cognitive resource) or 60 s (*Low time pressure*: high cognitive resource) to make their drink selection, these times were based on a previous online selection task [19] and an informal pilot [21]. Participants were informed of the time limit for making their selection at the start of the task and they were given a practice task in which they were asked to choose a snack from a selection. In the practice, participants were automatically moved on after the allocated time allowed. However, in the main selection task, participants had to make a choice before they could proceed to the next question (i.e., the time pressure was not enforced) to avoid missing data on the primary outcome. The amount of time taken to make the selection was recorded.

### Participants

Participants (*n* = 808) were recruited through the Prolific crowdsourcing platform [25]. Prolific pre-screening questions were used; therefore, the study was only advertised to members who met the inclusion criteria: aged 18+; regular alcohol consumer (i.e., at least weekly); regularly buy/drink beer; and UK resident. The study took approximately five minutes to complete and participants were reimbursed £0.75. Participants who failed an attention check question were excluded post-randomisation and replaced; they were not reimbursed as explained in the participant study information.

The sample size was calculated, using Stata, for a logistic regression model, with power of 0.8 and an alpha of 0.01, assuming an effect size with odds ratio (OR) of 2 and a reference probability of 0.34 based on a previous online study of healthier and less healthy snack availability [17], with a binomial distribution and balanced groups. This gave a sample size estimation of 404 for a two group comparison (i.e., 202 per group), and a total sample size of 808 for the four availability group comparisons.

### Measures

#### Primary outcome measure

The selection of a non-alcoholic drink was the primary outcome. The drinks presented were based on a previous online drink selection task [26].

#### Selection in relation to cognitive resource

A time pressure manipulation of eight seconds (*High time pressure*: low cognitive resource) or 60 s (*Low time pressure*: high cognitive resource) was used to examine the impact of cognitive resource on the selection of a non-alcoholic drink. Experience of time pressure was assessed using the following item based on a previous selection task [19]: “To what extent do you agree that you felt time pressure when making your drink choice?”, rated on a 7-point scale from “strongly disagree” to “strongly agree”.

#### Screening and demographic characteristics

Participants were asked their age, gender, residency (‘England’, ‘Wales’, ‘Scotland’, ‘Northern Ireland’, ‘Other (please specify)’, or ‘I do not live in the UK’) and highest qualification attained. The latter used Office for National Statistics categories for highest educational attainment based on UK educational qualifications or professional or vocational equivalents: ‘Higher Education or professional / vocational equivalents’ (e.g., post-school diploma, university degree), ‘A levels or vocational level 3 or equivalents’ (e.g., school exams age 18), ‘GCSE / O Level grade A*-C or vocational level 2 or equivalents’ (e.g., school exams age 16), ‘Qualifications at level 1 and below’ (e.g., essential work-based skills), ‘Other qualifications: level unknown’, or ‘No qualifications’ [27].

#### Thirst

Participants were asked about their current level of thirst before the drink selection task using a visual analogue (VAS) 0–100, from ‘not at all’ to ‘extremely’: “How thirsty are you feeling right now?”

#### Drinking behaviour risk

The Alcohol Use Disorders Identification Test (AUDIT) [28] questions were used to assess the level of risk associated with participants’ drinking behaviour.

#### Explanation of drink choice

Participants were asked to briefly explain why they chose the drink they selected via an open text box to inform future studies in this area.

#### Attention check

An attention check was embedded within the questions post-randomisation, given concerns regarding participants’ attention in unsupervised (i.e., online) settings: ‘When was the last time you flew to Mars?’ (‘never’; ‘a few days ago’; ‘weeks ago’; ‘months ago’). Only ‘never’ responses were considered satisfactory.

Participants were also shown an image of an alcohol-free beer after the selection task and asked to rate it based on liking, intention to purchase and consume [21]. These data have not been reported as they were not considered to contribute to understanding of availability interventions. However, the data can be accessed via data.bris (see ‘Availability of data and materials’).

### Procedure

The study was advertised to eligible Prolific members via their online account, which provided a link to the study on the Qualtrics platform. Participants were shown an information statement explaining the study, what they would be required to do, and informed that they could withdraw from the study by closing their browser. Before commencing the study, participants completed a tick-box consent page.

Participants first completed screening, thirst and demographic questions, those who did not meet the inclusion criteria were taken to the end of the experiment. Participants were then randomly assigned to one of eight conditions. Participants were shown a practice trial in which they were asked to select a snack they would like to eat today, followed by the main task in which they were asked to select one drink that they would like to drink today from a selection of non-alcoholic drinks and alcoholic beers depending on condition. Selection was made either under high time pressure or low time pressure, depending on condition. Participants were next asked about their experience of time pressure and then asked to briefly explain why they chose the drink they selected. Finally, participants rated a non-alcoholic drink and answered questions about their typical drinking behaviour. An attention check question was embedded within the study, and participants who provided unsatisfactory responses (see above) were excluded. After completing the study, participants were presented with a debriefing screen including information about how to find more information and contact the study team.

### Analysis

#### Primary objective: selection of a non-alcoholic drink

The odds of selecting a non-alcoholic drink when changing the absolute and / or relative availability of non-alcoholic drinks, while increasing the total number of all drinks, was calculated using a logistic regression model to compare the primary outcome between *Reference* 1 and: *Reference* 2; the *Increased non-alcoholic drinks* condition; and the *Increased alcoholic drinks* condition.

The odds of selecting a non-alcoholic drink, when changing the absolute and relative availability of non-alcoholic drinks, while holding the total number of all drinks constant, was calculated using a logistic regression model to compare the primary outcome between *Reference* 2 and: the *Increased non-alcoholic drinks* condition*;* and the *Increased alcoholic drinks* condition.

A trend analysis was also conducted comparing the *Increased non-alcoholic drinks* condition, *Reference* 2, and the *Increased alcoholic drinks* condition, using a logistic regression model with these three groups in order, as a continuous independent variable, i.e., *Increased alcoholic* condition (coded 1), *Reference* 2 (coded 2) and the *Increased non-alcoholic* condition (coded 3).

#### Secondary objective: selection in relation to cognitive resource

Logistic regression was used to assess the odds of selecting a non-alcoholic drink using a 4 (availability) × 2 (cognitive resource) design.

## Results

**Fig. 2:**
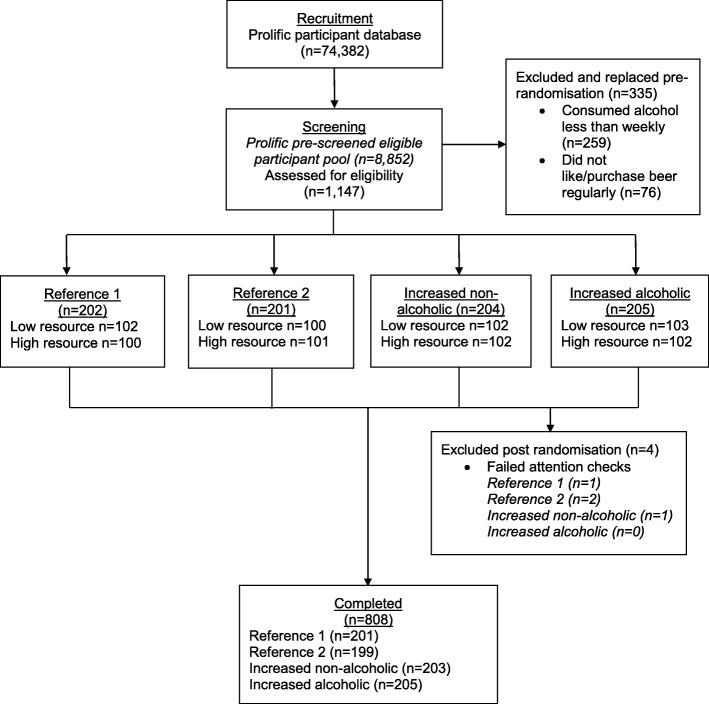
CONSORT diagram

A total of 1147 UK adults were assessed for eligibility and 812 participants were randomised to one of the eight conditions (see Fig. 2). Four (< 1%) participants were excluded post-randomisation because they failed the attention check; therefore, 808 participants were included in the analyses. The mean age was 38.2 years (SD = 12.2, range 18–75 years) and just over half of participants (*n* = 452, 56%) were male. The majority of participants reported higher education qualifications (Table 1). AUDIT scores suggested that, on average, participants reported ‘increasing risk’ levels of drinking; however, scores ranged 2–33 demonstrating very low risk drinking to possible dependence.

**Table 1:** Demographic characteristics of participants randomised to the four availability conditions

	*Reference* 1 (*n* = 201)	*Reference* 2 (*n* = 199)	*Increased non-alcoholic drinks* (*n* = 203)	*Increased alcoholic drinks* (*n* = 205)
Gender (%, n)				
*Male*	52 (105)	56 (112)	57 (115)	59 (120)
*Female*	48 (96)	43 (86)	42 (86)	41 (83)
*Other*	0	0	1 (2)	1 (2)
*Prefer not to say*	0	1 (1)	0	0
Age (M, SD)	38.2 (11.7)	37.6 (12.4)	38.8 (13.5)	38.0 (11.2)
Education level (%, n)				
*Higher education*	63 (126)	70 (139)	67 (135)	69 (142)
*A levels*	24 (48)	18 (36)	19 (38)	19 (38)
GCSE / O level A*-C	11 (23)	10 (20)	13 (26)	8 (16)
*Qualifications Level ≤ 1*	1 (2)	2 (3)	1 (1)	2 (4)
*Other qualifications*	0	1 (1)	1 (1)	2 (3)
*No qualifications*	1 (2)	0	1 (2)	1 (2)
Baseline thirst (M, SD)	49.3 (22.3)	52.5 (21.5)	48.0 (21.2)	51.2 (22.3)
AUDIT score (M, SD)	9.7 (5.6)	9.3 (5.1)	10.4 (6.0)	9.2 (4.8)

### Selection of a non-alcoholic drink

**Fig. 3:**
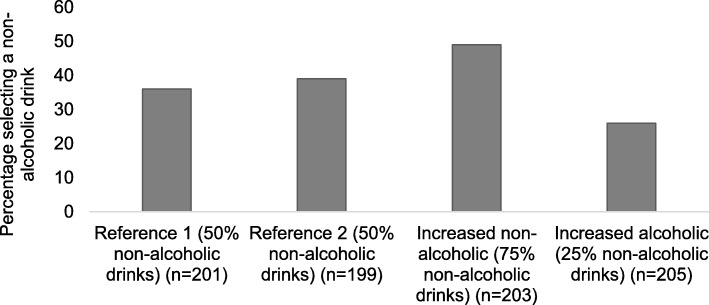
The proportion of participants selecting a non-alcoholic drink in the four availability conditions

Across the four conditions varying in non-alcoholic drink availability, the proportion selecting a non-alcoholic drink ranged from 26 to 49% (Fig. 3). The proportion of participants who selected a non-alcoholic drink was largest when non-alcoholic drink availability was increased (49%) and smallest when alcoholic drink availability was increased (26%) compared to equal availability of both options (*Reference* 1: 36% and *Reference* 2: 39%).

The logistic regression models provided evidence of an impact on selection of increasing non-alcoholic drink availability. Compared to *Reference* 1 (two non-alcoholic and two alcoholic drinks), non-alcoholic drink selection was similar in *Reference* 2 (four non-alcoholic and four alcoholic drinks) when the total number of drinks increased (absolute availability) but the proportion of non-alcoholic compared to alcoholic drinks (relative availability) was unchanged (OR = 1.15, 95% CI 0.77, 1.73) (Table 2). In contrast, the odds of selecting a non-alcoholic drink were 71% higher when both the absolute and relative availability of non-alcoholic compared to alcoholic drinks increased (*Increased non-alcoholic drinks:* six non-alcoholic and two alcoholic drinks) (OR = 1.71, 95% CI 1.15, 2.54). The odds of selecting a non-alcoholic drink were 37% lower when the relative availability of non-alcoholic drinks decreased (*Increased alcoholic drinks:* two non-alcoholic and six alcoholic drinks) (OR = 0.63, 95% CI 0.41, 0.96).

**Table 2:** Impact on non-alcoholic drink selection of non-alcoholic compared to alcoholic drink availability: Odds ratios of selecting a non-alcoholic drink, compared to i. Reference 1 and ii. Reference 2

	*Reference* 1 *(n = 201)*	*Reference* 2 *(n = 199)*	*Increased non-alcoholic (n = 203)*	*Increased alcoholic (n = 205)*
***OR (95% CI), p value***	[Reference]	1.15 (0.77, 1.73), *p* = 0.49	1.71 (1.15, 2.54), *p* < 0.01	0.63 (0.41, 0.96), *p* = 0.03
	–	[Reference]	1.48 (0.99, 2.19), *p* = 0.05	0.54 (0.35, 0.83), *p* < 0.01

Compared to *Reference* 2 (four non-alcoholic and four alcoholic drinks) – when the total number of all drink options remains constant – the odds of selecting a non-alcoholic drink were 48% higher when the relative availability of non-alcoholic drinks increased (*Increased non-alcoholic drinks:* six non-alcoholic and two alcoholic options) (OR = 1.48, 95% CI 0.99, 2.19), and were 46% lower when their relative availability decreased (*Increased alcoholic drinks:* two non-alcoholic and six alcoholic drinks) (OR = 0.54, 95% CI 0.35, 0.83). In addition, a trend analysis compared the odds of selecting a non-alcoholic drink when increasing their relative proportion: for every increase, there was evidence of an increase in the proportion of non-alcoholic drinks selected (OR = 1.64, 95% CI 1.34, 2.02, *p* < 0.001).

Sensitivity analyses were conducted with adjustments made to: *i.* include participants who failed the attention check (*n* = 4), and *ii.* account for baseline thirst. The results of the primary analyses were not substantially affected.

### Cognitive resource

An initial logistic regression model with two main effects of availability and cognitive resource showed no evidence of an interaction effect (*p* = 0.98); therefore, the interaction term was dropped in favour of a model which included only the two main effects. There was no evidence of a difference in selection of non-alcoholic drink between the *High* (37%, *n* = 403) and *Low* (38%, *n* = 405) conditions (OR = 0.96, CI 95% 0.72, 1.29, *p* = 0.79). Following the selection task, participants were asked whether they experienced time pressure: 80% of those in the eight-second condition agreed that they did, compared to 31% in the 60-s condition.

A sensitivity analysis was conducted excluding participants who were randomised to the select under high time pressure but who took more than eight seconds to make a selection (*n* = 48, 12%) and the results of the original analysis were not substantially affected (OR = 0.96 95% CI 0.72, 1.30, *p* = 0.81).

## Discussion

To our knowledge, this online study provides the first evidence that increasing both the absolute and relative availability of non-alcoholic drinks can increase their selection, compared to alcoholic drinks, in keeping with the study hypothesis. Similarly, decreasing the relative availability of non-alcoholic drinks can reduce their selection compared to alcoholic drinks.

These findings are consistent with existing evidence showing an effect on selection and consumption of snacks and meals of altering healthier and less healthy food options (10). The proportion of participants in the current study selecting the healthier non-alcoholic drink – compared to the less healthy alcoholic drink – was similar to those selecting a healthier lower calorie snack, compared to a less healthy high calorie snack, in a similar online selection task [17] across availability conditions: equivalent healthier and less healthy options (36 and 39% non-alcoholic drinks, 38% low calorie snacks); increased healthier options (49% non-alcoholic drinks, 55% low calorie snacks); increased less healthy options (26% non-alcoholic drinks, 12% low calorie snacks). These results suggest that availability interventions to encourage healthier selection, in this case choosing non-alcoholic rather than alcoholic drinks, may be most effective when changing the relative availability of options, i.e., increasing the proportion of non-alcoholic drinks and consequently decreasing the proportion of alcoholic drinks available for selection.

Awareness of these alternatives among consumers is necessary for their selection. The proportion of participants selecting a non-alcoholic drink in the present study was under half across all availability conditions, even when 75% of options displayed were non-alcoholic. In real-world settings, such as bars and restaurants, non-alcoholic drink options are far more limited and often lack visibility, for example, by being displayed in the bottom of a fridge behind a bar. Some venue guides have been generated to support reduced alcohol consumption, e.g., Club Soda [29], to overcome the difficulties identifying drink options in the face of pervasive alcohol marketing in busy drinking environments, and stigma regarding non-alcoholic drink selection and consumption [30]. Although alcohol-free beer, wine and spirit alternatives currently make up a very small proportion of the market, this is growing [31], and improving the selection and promotion of non-alcoholic drinks provides an opportunity for licensed venues to reduce alcohol consumption without losing revenue. Increasing the availability of non-alcoholic drink options could increase their salience and make it easier for consumers to identify alternatives, as well as shifting expectations and norms in relation to seeing, purchasing and consuming non-alcoholic drinks in social settings over the longer term.

The present study also examined the role of cognitive resource (*High* or *Low*) on drink selection and found no effect, which was consistent across all four availability conditions. It is possible that the time pressure manipulation did not reliably reduce cognitive resource, particularly given the online setting, which is likely to have been free from other cognitive burdens that might be present in real-world drinking situations. However, our results are consistent with a previous online snack selection task, which found no effect of cognitive resource on selection when using a digit recall task (complex or simple) [17]. Limitations were raised in the snack study regarding the possibility that participants could have avoided the recall task in the online setting. However, the use of the time pressure manipulation mitigated this concern, and the manipulation check suggested that participants did experience the time pressure as expected. Therefore, it may be more likely that the results reflect an effect on selection of increasing non-alcoholic drink availability that operates regardless of cognitive resource, which would support the implementation of availability interventions in real-world settings.

### Strengths and limitations

This study provides, to our knowledge, the first evidence that altering the absolute and relative availability of non-alcoholic and alcoholic drink options can impact their selection. Evidence-based policy recommendations to reduce alcohol harm [32] include incentivising the development of low and non-alcoholic drink products, increasing the choice for consumers, as part of a broader public health strategy. The promotion of non-alcoholic alternatives presents an opportunity for licensed venues to address licensing objectives to promote public safety and prevent crime and disorder [33] through less punitive measures [30]. However, it is important to note that this online study measured hypothetical selection. The participants were recruited through one online platform (Prolific), and while the study was made available to all eligible participants, engagement may not represent a random sample of these participants. Evidence is now required to determine whether similar effects are observed in field or lab settings involving selection of an actual drink, which would provide greater external validity of results. In addition, the current study sample were unrepresentative of the general population in being highly educated, with 67% of the current sample having degree level qualifications or higher, compared to 27% of residents in England and Wales aged 16+ (some people in the census had not completed education) [34]. Although the current study was not powered to explore differences in selection according to education level, the impact on snack selection of healthier option availability was previously found to be consistent across socioeconomic groups [17]. However, future studies should consider whether availability may differentially impact selection according to socioeconomic factors.

## Conclusions

Greater availability of non-alcoholic drinks, compared to alcoholic drinks, increased their online selection, an effect that may be larger when changing their relative availability, i.e., increasing the proportion of non-alcoholic drinks. Studies in real-world settings are needed to determine the impact of availability interventions on reducing purchasing and consumption of alcoholic drinks.

## Data Availability

The anonymised data collected are available as open data via the University of Bristol online data repository: 10.5523/bris.1dnhjcw6w4m1n29u2yuxjywde1.

## Abbreviations

UK: United Kingdom
TIPPME: Typology of Interventions in Proximal Physical Micro-Environments
OR: odds ratio
GCSE: General Certificate of Secondary Education
O level: General Certificate of Education Ordinary Level
A level: General Certificate of Education Advanced Level
VAS: visual analogue scale
AUDIT: Alcohol Use Disorders Identification Test
SD: standard deviation
CI: confidence interval
M: mean
